# Pirt contributes to uterine contraction-induced pain in mice

**DOI:** 10.1186/s12990-015-0054-x

**Published:** 2015-09-17

**Authors:** Changming Wang, Zhongli Wang, Yan Yang, Chan Zhu, Guanyi Wu, Guang Yu, Tunyu Jian, Niuniu Yang, Hao Shi, Min Tang, Qian He, Lei Lan, Qin Liu, Yun Guan, Xinzhong Dong, Jinao Duan, Zongxiang Tang

**Affiliations:** College of Basic Medicine, Nanjing University of Chinese Medicine, 138 Xianlin Rd, Nanjing, 210023 Jiangsu China; College of Life Science, Nanjing Normal University, Nanjing, 210046 Jiangsu China; Department of Anesthesiology, University of Washington, St. Louis, MO 63110 USA; Department of Anesthesiology and Critical Care Medicine, School of Medicine, Johns Hopkins University, Baltimore, MD 21205 USA; The Solomon H. Snyder Department of Neuroscience, Center for Sensory Biology, School of Medicine, Johns Hopkins University, Baltimore, MD 21205 USA

**Keywords:** Pirt, Uterine contraction-induced pain, Dorsal root ganglion, TRPV1

## Abstract

Uterine contraction-induced pain (UCP) represents a common and severe form of visceral pain. Nerve fibers that innervate uterine tissue express the transient receptor potential vanilloid channel 1 (TRPV1), which has been shown to be involved in the perception of UCP. The phosphoinositide-interacting regulator of TRP (Pirt) may act as a regulatory subunit of TRPV1. The intraperitoneal injection of oxytocin into female mice after a 6-day priming treatment with estradiol benzoate induces writhing responses, which reflect the presence of UCP. Here, we first compared writhing response between *Pirt*^+*/*+^ and *Pirt*^**−/−**^ mice. Second, we examined the innervation of Pirt-expressing nerves in the uterus of *Pirt*^**−/−**^ mice by immunofluorescence and two-photon microscopy. Third, we identified the soma of dorsal root ganglion (DRG) neurons that innerve the uterus using retrograde tracing and further characterized the neurochemical properties of these DRG neurons. Finally, we compared the calcium response of capsaicin between DRG neurons from *Pirt*^+*/*+^ and *Pirt*^**−/−**^ mice. We found that the writhing responses were less intensive in *Pirt*^**−/−**^ mice than in *Pirt*^+*/*+^ mice. We also observed Pirt-expressing nerve fibers in the myometrium of the uterus, and that retrograde-labeled cells were small-diameter, unmyelinated, and Pirt-positive DRG neurons. Additionally, we found that the number of capsaicin-responding neurons and the magnitude of evoked calcium response were markedly reduced in DRG neurons from *Pirt*^−*/*−^ mice. Taken together, we speculate that Pirt plays an important role in mice uterine contraction-induced pain.

## Background

Study of uterine contraction-induced pain (UCP) that occurs during labor or menstruation (dysmenorrhea) has received little attention compared with the study of other visceral pain conditions. The uterus is innervated by afferent hypogastric nerve fibers through which any sensory information is transmitted to the central nervous system (CNS) [1]. Although the hypogastric nerve also transmits noxious mechanical and chemical stimuli, pain induced by uterus cervical distension shows similarity to the heat hyperalgesia observed in an animal models [2]. This similarity suggests a potential role of heat-sensing ion channels in pain induced by cervical and uterine contraction. Indeed, the transient receptor potential vanilloid 1 (TRPV1), which is important to the detection of noxious heat, may contribute to the perception of visceral pain [3–6].

Previous studies have also suggested an important role of estrogen in the modulation of visceral pain. Sex hormones may underlie gender differences in pain perception and in analgesia [7]. Estrogen was suggested to contribute to visceral pain hypersensitivity and to the increased sensitivity to visceral pain in animal models [2, 8–10]. Moreover, estrogen may amplify pain responses to uterine cervical distension in animal models by enhancing TRPV1 function [11]. Estrogen not only increased TRPV1 expression in afferent sensory neurons innervating the uterus [10], but also exaggerated the uterine pain evoked by capsaicin and heat stimulation [11]. On the other hand, the activation of nerve fibers in the uterine horn was reversed by intrauterine pretreatment with capsazepine, a TRPV1-selective antagonist [11, 12]. The level of TRPV1 expression in ectopic endometrium is also correlated with the severity of dysmenorrhea in patients [13]. These findings suggest an important role of TRPV1 in uterine pain perception [12].

The phosphoinositide-interacting regulator of TRP (Pirt) is a transmembrane protein that is expressed in most nociceptive neurons in the dorsal root ganglia (DRG), but not in CNS. Pirt has recently been identified as a key component of the TRPV1 and TRPM8 complexes and acts as an endogenous enhancer of TRPV1 and TRPM8 function, [14, 15]. Since TRPV1 participated in UCP [10, 11, 14], we hypothesized that Pirt may also play an important role in the perception of UCP by interacting with TRPV1. In the present study, we demonstrated that Pirt was involved in the perception of UCP.

## Methods

### Animals

This study was approved by the Animal Care and Use Committee of Nanjing University of Chinese Medicine (Nanjing, China). Experiments were conducted according to the animal research ethical guidelines of the International Association for the Study of Pain. Female C57BL/6 mice were housed in groups of four per cage in our GLP animal center, with free access to food and water.

*Pirt*^**−/−**^ mice were bred in the laboratory of one of the authors (XD). For the generation of *Pirt*^−*/*−^ mice, the entire Pirt coding region was replaced with an EGFPf-IRESrtTA-ACN- targeting construct to produce a null allele [14]. Offspring *Pirt*^−*/*−^ and *Pirt*^+*/*+^ littermates were generated by breeding heterozygotes. Only healthy animals weighing 15–20 g and displaying normal water and food intake were included in the study.

### Behavior analysis

Animal behavior experiments were performed in a controlled environment of 20–24 °C, 45–65 % humidity, and a 12-h day/night cycle. Female *Pirt*^+*/*+^ and *Pirt*^−*/*−^ mice (5–6 weeks old) were used in all experiments. Animals were acclimated to the testing environment for 30 min before the initiation of writhing behavior tests. Estradiol benzoate was administered by intraperitoneal (i.p.) injection daily at 9 a.m. for 6 consecutive days (0.01 g/kg/day). One day after estradiol priming (i.e., day 7), oxytocin was injected (0.01 L/kg, i.p.) to induce uterine contractions. Writhing behavior responses were recorded with a digital video camera (HDR-PG820E, Sony, Minato, Tokyo, Japan) for 80 min after oxytocin injection. Abdominal writhing was defined as an exaggerated extension of the abdomen combined with the outstretching of the hindlimbs [16]. Animal behavior (e.g., the number of writhing motions) was analyzed by investigators who were blind to animal treatment conditions.

### Real-time PCR

Total RNA was extracted from freshly isolated DRG using TRIzol reagent (Invitrogen) and treated with RQ1 Dnase (Promega). The reverse transcription was performed using the Transcript First Strand cDNA Synthesis kit (Roche, Basel, Switzerland).

For qPCR, Light Cycler 480 SYBR Green I Master (Roche, Basel, Switzerland) was used. The reaction was run in an Lihgt Cycler 480 II Real-Time PCR instrument (Roche, Basel, Switzerland) using 1 μL of the cDNA in a 20 μL reaction according to the manufacturer’s instructions. The sequences of the mouse Pirt primers were as follows: forward primer: TAGACGAGAGGTCTCCAGAGT; reverse primer: CCAGTTGCTTTTGGGTGTGG. The sequence of mouse GAPDH primers were as follows: forward primer: ACCACAGTCCATGCCATCAC; reverse primer: TCCACCACCCTGTTGCTGTA. Calibrations and normalizations were done using the following 2^−∆∆CT^ method: where ∆∆C_T_ = (CT (target gene) − CT (reference gene)) − (CT (calibrator) − CT (reference gene)). GAPDH was used as the reference gene for qPCR experiments.

### Uterine tissue extraction and H&E-staining

For uterine tissue retrieval, mice were anesthetized with 1 % sodium pentobarbital (50 mg/kg, i.p.), and perfused transcardially with 10–15 mL phosphate-buffered saline (PBS) followed by 10–15 mL of ice-cold 4 % paraformaldehyde (pH 7.4). We performed a laparotomy via a lower abdomen midline incision to dissect and remove the uterus. Then we post-fixed the uterus in 4 % paraformaldehyde in PBS for 3 h and stored it overnight in 30 % sucrose for cryoprotection. The uterus was then embedded in optimum cutting temperature compound (OCT, Leica, Wetalar, Germany) and rapidly frozen at −20 °C (CM1950, Leica). Cryoembedded tissues were cut into 10-μm thick slices on a sliding microtome (CM1950, Leica). Conventional H&E-staining technique was used to confirm the distribution of Pirt nerve fiber terminals on the uterus. All imaging was performed with an Olympus fluorescence microscope (BX51, Olympus, Shinjuku, Tokyo, Japan).

### Detecting Pirt-expressing nerve fibers in the uterus

We used fluorescence microscopy to examine Pirt-expressing nerve fibers in the uterus of *Pirt*^−*/*−^ mice. Because of the EGFPf-IRESrtTA-ACN-targeting construct in *Pirt*^−*/*−^ mice, Pirt-expressing neurons and nerve fibers can be identified by green fluorescence, the expression of which is driven by the endogenous Pirt-promoter gene. Adjacent uterus sections were stained with hematoxylin and eosin (H&E), and overlapping fluorescence (Pirt) with bright-field (H&E) images allowed us to identify the histological site of Pirt-expressing nerve fibers in the uterus. Images were acquired and overlapped by using stereological software (Stereo Investigator 10, MBF, Williston, VT, USA).

### Two-photon microscopy imaging

Uteruses from *Pirt*^−*/*−^ mice were also examined by using two-photon imaging. Tissue was prepared as for immunofluorescence. In brief, the uterus was cut into 0.5–1.0 cm length tissue and fixed in 4 % paraformaldehyde, then moved to 30 % sucrose solution overnight. Tissues were removed from the sucrose solution and cut into 100-μm thick sections after precipitation. The section was moved to the stage of two-photon fluorescence microscope; 920 nm excitation wavelength was chosen. Image was captured at every 1 nm thickness with a 25× hydroscope and restructured after 100 shots (FV-1000, Shinjuku, Tokyo, Japan). The results of the imaging were overlapped with the results of H&E staining.

### Retrograde tracing of DRG neurons that innervate the uterus

*Pirt*^+*/*+^ and *Pirt*^−*/*−^ mice were anaesthetized with 1 % sodium pentobarbital (40 mg/kg, Merck, Darmstadt, Germany) and restrained in a supine position. A laparotomy was performed via a lower abdomen midline incision, and the uterus was dissected from the bladder. Then, 30 μL of 1,1′-Dioctadecyl-3,3,3′,3′-tetramethylindocarbocyanine perchlorate (Dil, 0.25 % in DMSO, Sigma-Aldrich, St. Louis, MO, USA) was gradually instilled into the myometrium and perimetrium through a 50-μL Hamilton syringe. Different injection sites were chosen randomly in the cervix and body, and each site was swabbed with fresh cotton balls to absorb leaking tracer. After the dye injection, the peritoneal cavity was rinsed with warm saline and the wound was sutured shut. The animals were positioned supine during injection and kept in that position until they recovered from anesthesia. All surgical procedures were performed under sterile conditions. Immunofluorescence and calcium imaging experiments were conducted in mice at 14 days after the dye injection. DRG from spine levels T10–L5 were cut into 10-μm slices for examination of the retrograde-labeled cells. To calculate the Dil labeled cells in every DRG, every two slices were captured and counted.

### Immunostaining of DRG neurons

DRG tissues were collected from spine levels T10–L5, and were then used to immunostain for neurofilament protein-200 (NF-200), plant isolectin B_4_ (IB_4_), and TRPV1. For immunostaining, we incubated sections in blocking solution (containing 3 % fetal bovine serum, 0.1 % Triton X-100, and 0.02 % sodium azide in PBS) for 2 h at room temperature and then at 4 °C overnight with mouse anti-NF-200 (1:1000; Invitrogen, Grand Island, NY, USA) and isolectin IB_4_ Alexa Fluor dye conjugate (1:1000; Invitrogen) primary antibodies. Next, the sections were incubated in Alexa Fluor-conjugated goat anti-mouse IgG (1:100, Earthox, Millbrae, CA, USA) secondary antibody at room temperature for 2 h. Dil-labeled sections were also incubated with rabbit anti-TRPV1 (1:1000; Neuromics, Edina, MN, USA) primary antibodies at 4 °C overnight. Then, the sections were incubated in Alexa Fluor-conjugated donkey anti-rabbit IgG (1:100, molecular probes, Eugene, OR, USA) secondary antibody at room temperature for 2 h. By immunostaining DRG neurons, we counted the number of DRG neurons innervating the uterus, and also measured the diameter of stained neurons using Stereo Investigator 10 software. After each stained picture was captured, it was merged into an image. These neurons of Dil-labeled and fluorescently stained from the same slice were analyzed by using Stereo Investigator 10 analysis software.

### Cell culture

Fourteen days after the retrograde tracing procedure, the mice were anesthetized with 1 % sodium pentobarbital. The DRG from spine levels T10–L5 were harvested as previously described [10] and immediately transferred to cold DH10 medium (DMEM/F-12, 10 % FBS, 1 % penicillin–streptomycin-glutamine; Invitrogen). DRGs were washed 2–3 times in warm DH10 and then treated with enzyme solution (5 mg/mL dispase and 1 mg/mL collagenase type I in Hanks Balanced Salt Solution without Ca^2+^ and Mg^2+^, Invitrogen) at 37 °C until the cells dissociated [14, 17]. Dissociated cell suspensions were filtered through a 100-μm cell strainer (BD, Franklin Lakes, NJ, USA). After being centrifuged at 1500 rpm for 5 min, DRG neurons were resuspended in DH10, and nerve growth factor was added (50 ng/mL, Millipore, Billerica, MA, USA). Fifty microliters of suspended cells in solution were plated onto pre-sterilized glass coverslips that had been coated with 0.5 mg/mL poly-d-lysine (Biomedical Technologies, Inc., Stoughton, MA, USA) and 10 μg/mL laminin (Invitrogen). Plated neurons were cultured in an incubator (95 % O_2_ and 5 % CO_2_) at 37 °C and used for calcium imaging studies within 48 h.

### Calcium imaging

The DRG neurons were loaded with fura-2-acetomethoxy ester (Molecular Probes, Eugene, OR, USA) for 30 min at 37 °C in the dark in accord with previous studies [14, 17]. After being washed 3 times with PBS, the glass coverslips were placed into a chamber and perfused with a solution containing 137 mM NaCl, 5.4 mM KCl, 1.2 mM MgCl_2_, 1.2 mM NaH_2_PO_4_, 1 mM CaCl_2_, 10 mM glucose, and 20 mM HEPES (pH 7.4). A high-speed continuously scanning monochromatic light source (Polychrome V, Till Photonics, Gräfeling, Germany) was used for excitation at 340 and 380 nm, enabling us to detect changes in intracellular free calcium concentration.

### Data analysis

The methods for statistical comparison in each study are given in the Results section. The number of animals used in each study was based on our experiences and on similar studies. We grouped seven pairs of female *Pirt*^+*/*+^ and *Pirt*^−*/*−^ littermates for use in the experimental group and the control group in the behavioral experiments. The gynotype of mice was blinded to experimenters to reduce selection and observation bias. After the experiments were completed, no data point was excluded. Paired sample *t* tests (Spass 16.0) were used to compare the behavioral results. For morphological results, we used representative data from three mice with similar results. Statistical analysis of the number of Dil-labeled cells, Pirt-positive cells, and IB_4_-binding cells were carried out manually. We used representative data from Ca^2+^ imaging studies that were replicated at least 15 times from 3 mice (5 times in each mouse). We used independent sample *t* tests (Spass 16.0) to calculate the proportion of cells that responded and the intensity of response. *P* < 0.05 was considered statistically significant in all tests.

## Results

### *Pirt*^−*/*−^ mice exhibit less UCP than *Pirt*^+*/*+^ mice

**Fig. 1:**
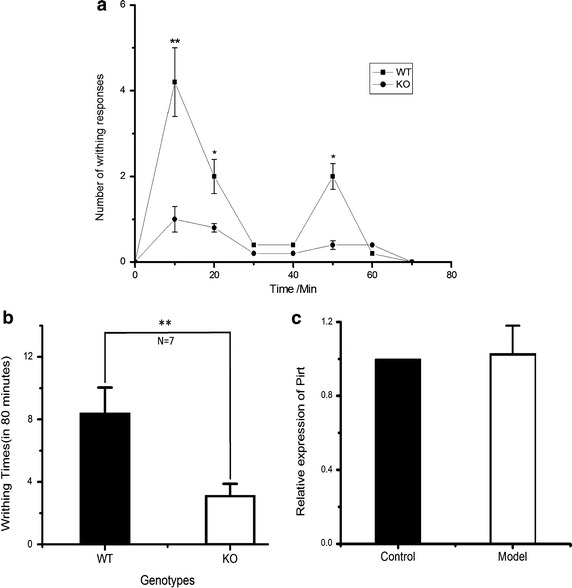
The writhing response behavior and gene expression analysis. **a** The time course of writhing response during the 80 min after oxytocin injection. **b** Number of writhing responses in *Pirt* ^+*/*+^ (8.4 ± 1.6) and *Pirt* ^−*/*−^ *mice* (3.1 ± 0.7) after 6 days of priming with estradiol benzoate and induction by oxytocin (^**^ *P* < 0.01, n = 7/group). **c** Real-time PCR showed that *Pirt* gene expression was not affected by treatment with estradiol and oxytocin

To investigate the role of Pirt in UCP, we first examined writhing behavior induced by intraperitoneal injection of oxytocin in *Pirt*^+*/*+^ and *Pirt*^−*/*−^ female mice after 6 days of priming with estradiol benzoate (n = 7/group). UCP in mice can be inferred by the stretching of the posterior limb followed by an abdominal contraction [16, 18, 19]. The number of writhing responses during the 80 min after oxytocin injection was significantly less in *Pirt*^−*/*−^ mice (3.1 ± 0.7) than in *Pirt*^+*/*+^ mice (8.4 ± 1.6, *P* < 0.01, paired *t* test Spass 16.0; Fig. 1b). The writhing responses often occurred in two phases (1st phase: 0–30 min; 2nd phase: 40–60 min after oxytocin injection, Fig. 1a), and subsided after 80 min. The decreased number of writhing responses in *Pirt*^−*/*−^ mice suggests an involvement of Pirt in UCP. The expression levels of Pirt were detected in UCP and normal mice DRG neurons by real-time PCR. We found no changes of Pirt-expressing in UCP (p > 0.05, n = 4, Fig. 1c).

### Pirt-expressing nerve fibers innervate the uterus

**Fig. 2:**
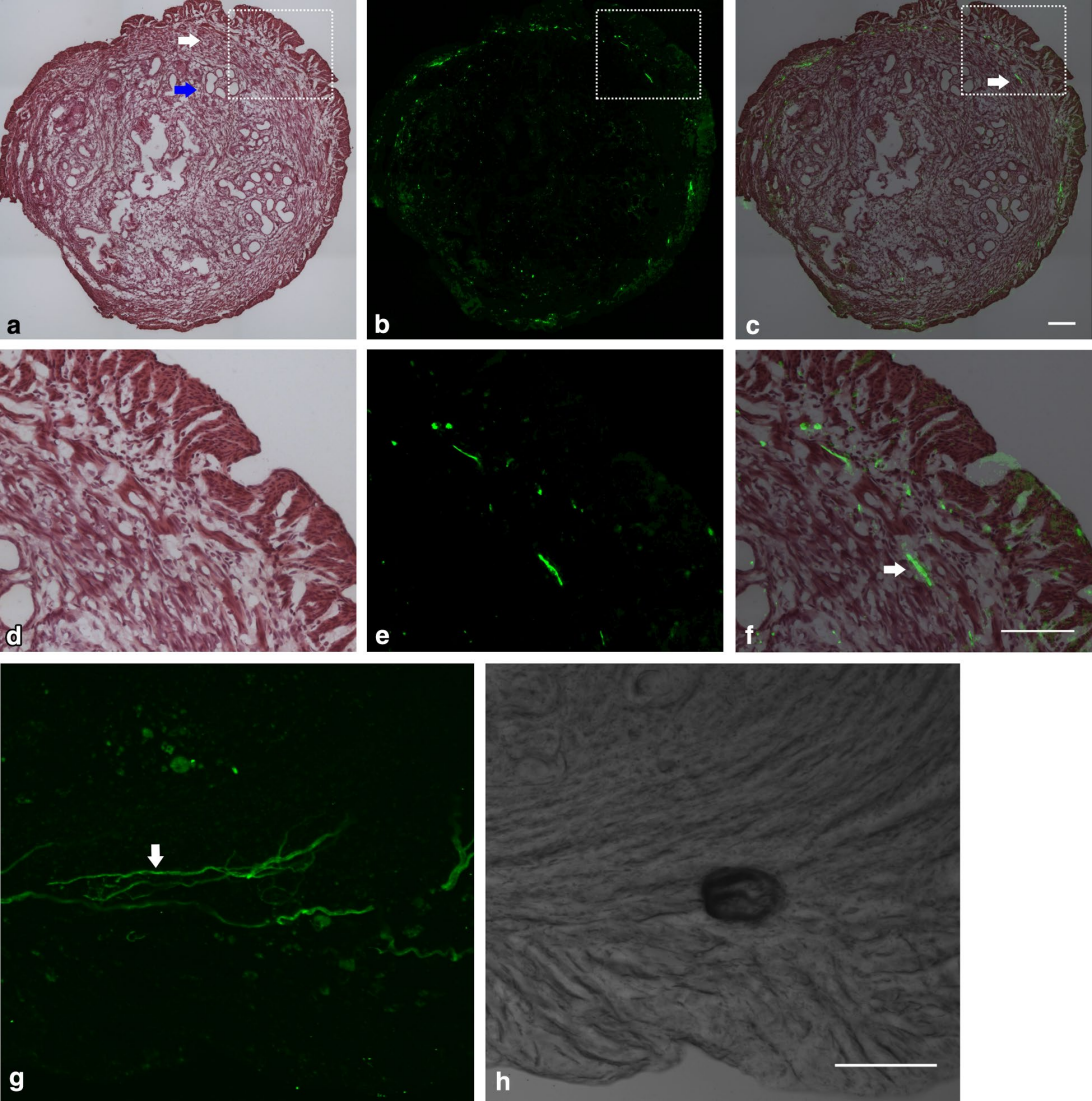
Pirt-expressing nerves innervate the uterus. **a** Hematoxylin and eosin-stained section of the uterus. Myometrium and endometrium are indicated by *white arrow* and *blue arrow*, respectively. **b** Image of the uterine section under fluorescence microscopy. In Pirt^−/−^ mice, the entire Pirt-coding region was replaced by an EGFPf-IRESrtTA-ACN-targeting construct, which expresses green fluorescent protein. Thus, Pirt-expressing fibers can be identified by *green fluorescence*. **c** Superimposed images of **a** and **b**. Pirt-expressing fiber is indicated by a *white arrow*. **d**–**f** Higher resolution views of the *boxed areas* in **a**–**c**, respectively. Fluorescence (**g**) and bright-field (**h**) images from two-photon microscopy analysis showing Pirt-expressing nerve fibers in the myometrium (*white arrow*). *Scale bar* 100 µm

Pirt is specifically expressed in DRG neurons in the peripheral nervous system [14]. As shown above, *Pirt*^−*/*−^ mice exhibited a less intensive writhing response than that of *Pirt*^+*/*+^ mice (Fig. 1), which led us to speculate that Pirt-expressing nerve fibers may innervate the uterus. In *Pirt*^−*/*−^ mice, the Pirt-coding region was replaced with a green fluorescent protein (GFP)-expressing construct (Fig. 2a–f). Thus, Pirt-expressing DRG neurons and their branches can be visualized by GFP fluorescence. By superimposing the bright-field H&E-staining over the fluorescence image of the same section, we found Pirt-expressing nerve fibers distribution in the myometrium of uterus (Fig. 2d–f). Subsequent two-photon microscopy imaging confirmed this finding (Fig. 2g–h).

### DRG neurons innervating the uterus are Pirt-positive

**Fig. 3:**
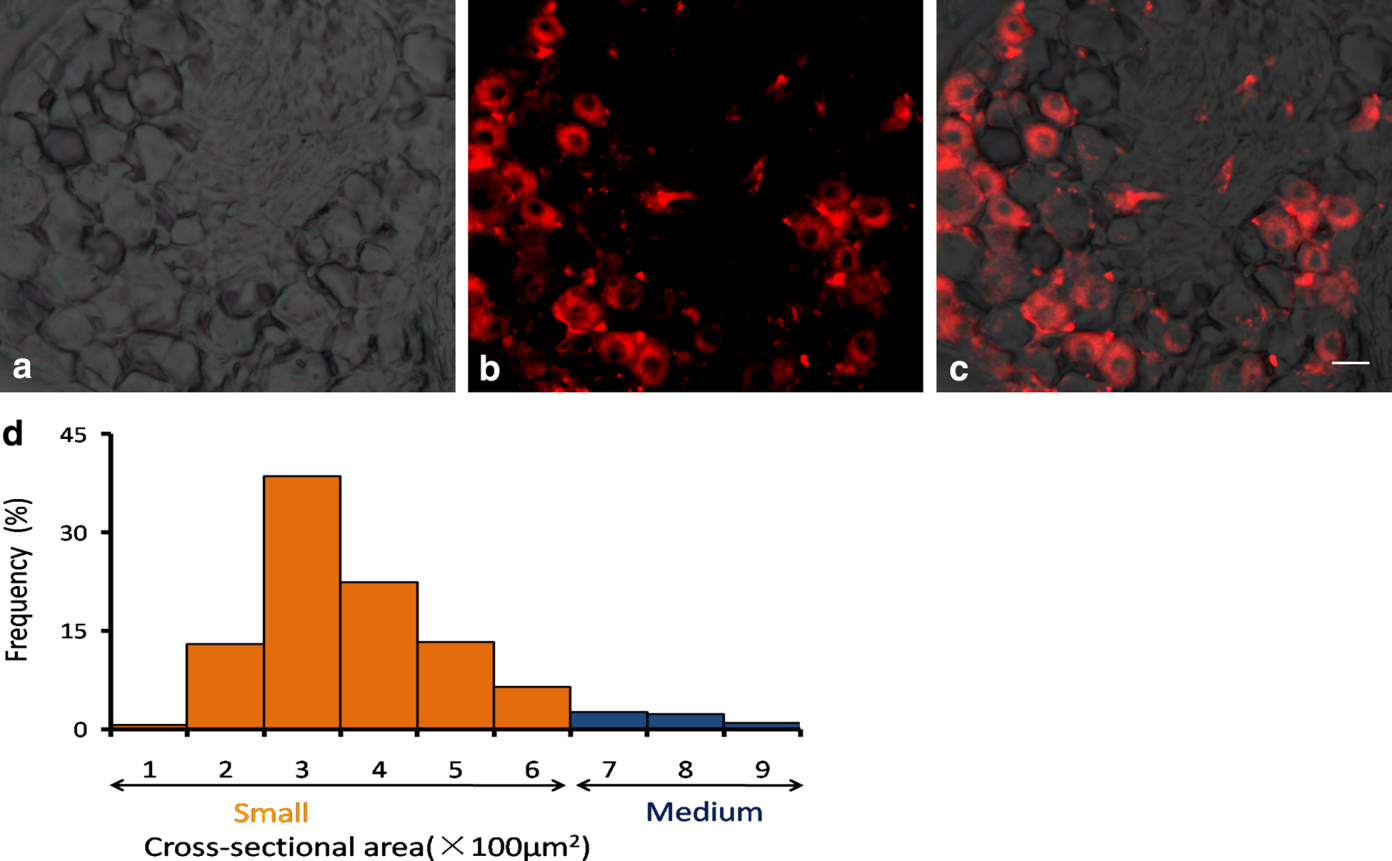
Small-diameter DRG neurons innervate the uterus. Bright-field (**a**) and fluorescence (**b**) imaging of Dil-labeled DRG. **c** Overlapping images from **a** and **b** permit differentiation between traced and untraced fibers. *Scale bar* 20 µm. **d** Most DRG neurons that received input from the uterus had a small soma size, with a surface area ranging from 100 to 600 μm^2^ (1599 labeled neurons in T13 DRG)

**Fig. 4:**
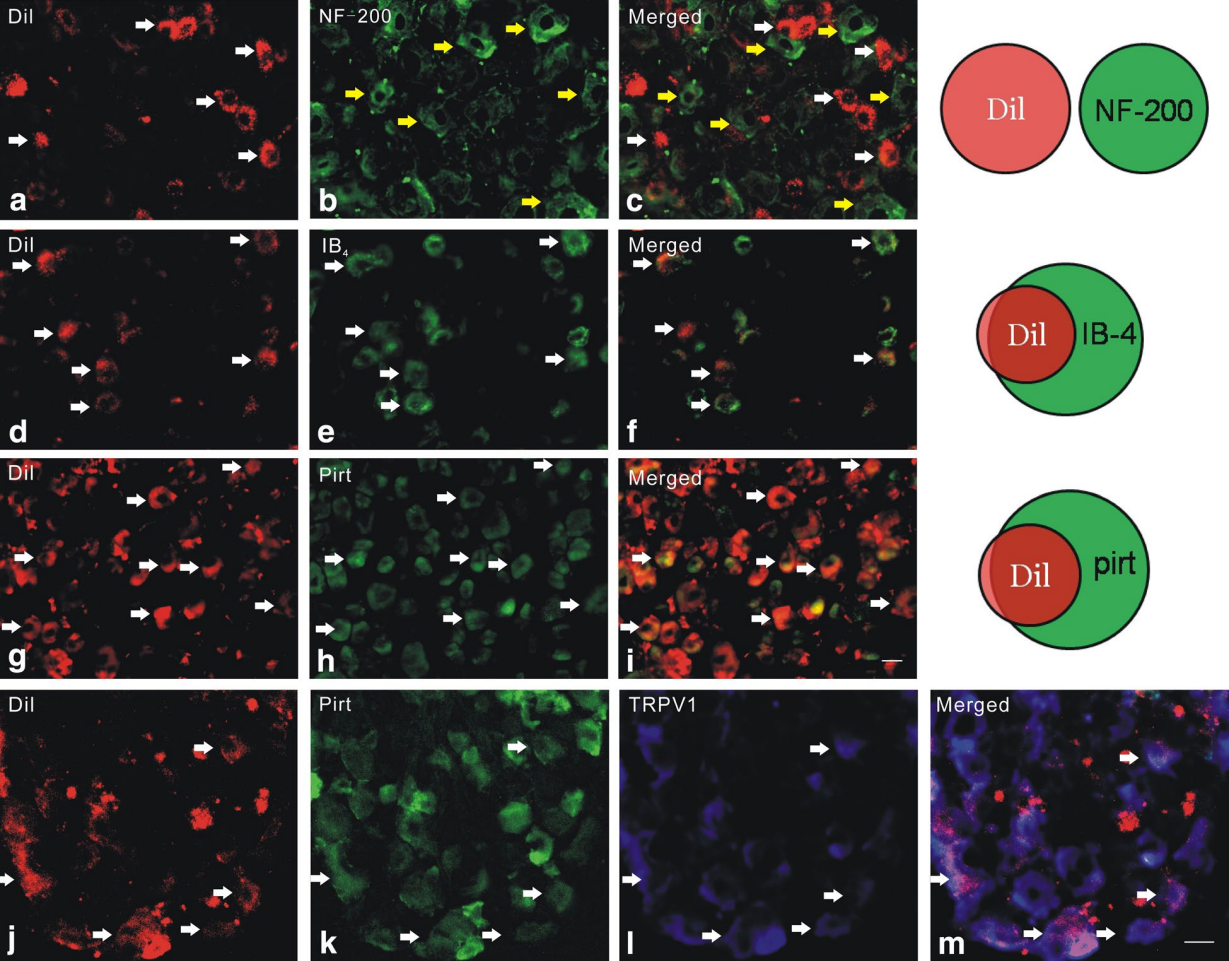
The neurochemical properties of Dil-labeled DRG neurons. **a**–**c** Dil-labeled cells did not colocalize with NF-200. Dil-labeled cells and NF-200-expressing cells are indicated by *white* and *yellow arrows*, respectively. Dil-labeled DRG neurons colocalized with IB4 (**d**–**f**) and Pirt (**g**–**i**). **j**–**l** DRG section including Pirt-positive and Dil-labeled neurons was immunostained by TRPV1. **m** A merged image of Dil-labeled, Pirt-positive and TRPV1-staining neurons. The *white arrows* designate double-labeled cells. Pirt expression was assessed by fluorescence microscopy in Pirt^−/−^ mice, in which the entire Pirt-coding region has been replaced with an EGFPf-IRESrtTA-ACN-targeting construct. *Scale bar* 20 µm

Next, we further characterized the neurochemical properties of DRG neurons projecting to the uterus using retrograde labeling. Fourteen days after injection of Dil into the uterus, DRGs from thoracic and lumbar levels were isolated and examined given that hypogastric nerves innervating the uterine cervix and uterus originate primarily from T12–L2 DRGs [1, 10, 20]. We differentiated Dil-traced fibers from untraced fibers by overlapping Dil-labeling with bright-field images (Fig. 3c). The number of Dil-labeled DRG neurons at each level was as follows: T10, 148 ± 23; T11, 213 ± 21; T12, 358 ± 40; T13, 533 ± 46; L1, 263 ± 35; L2, 330 ± 28; L3, 127 ± 17; L4, 102 ± 14; and L5, 98 ± 16 (n = 3). Because the number of Dil-labeled cells was highest at T13, we thus further measured the size of Dil-labeled DRG neurons at this level. Most Dil-labeled DRG neurons were small-to-medium size with a surface area ranging from 100 to 600 μm^2^ (Fig. 3d). Immunostaining revealed that Dil-labeled neurons were not overlapped with NF200 stained neurons (Fig. 4a–c); 96.4 % of Dil-labeled DRG neurons were IB4-binding neurons (Fig. 4d–f) and 96 % of Dil-labeled DRG neurons were Pirt-positive (Fig. 4g–i). By double staining, 25 % of Pirt-expressing cells were TRPV1 positive, 46 % of Pirt-expressing cells were Dil-labeled, 31 % of Dil-labeled cells were TRPV1 positive (Fig. 4j–m).

### The percentage of capsaicin-response DRG neurons is more in *Pirt*^+*/*+^ mice than in *Pirt*^−*/*−^ mice

**Fig. 5:**
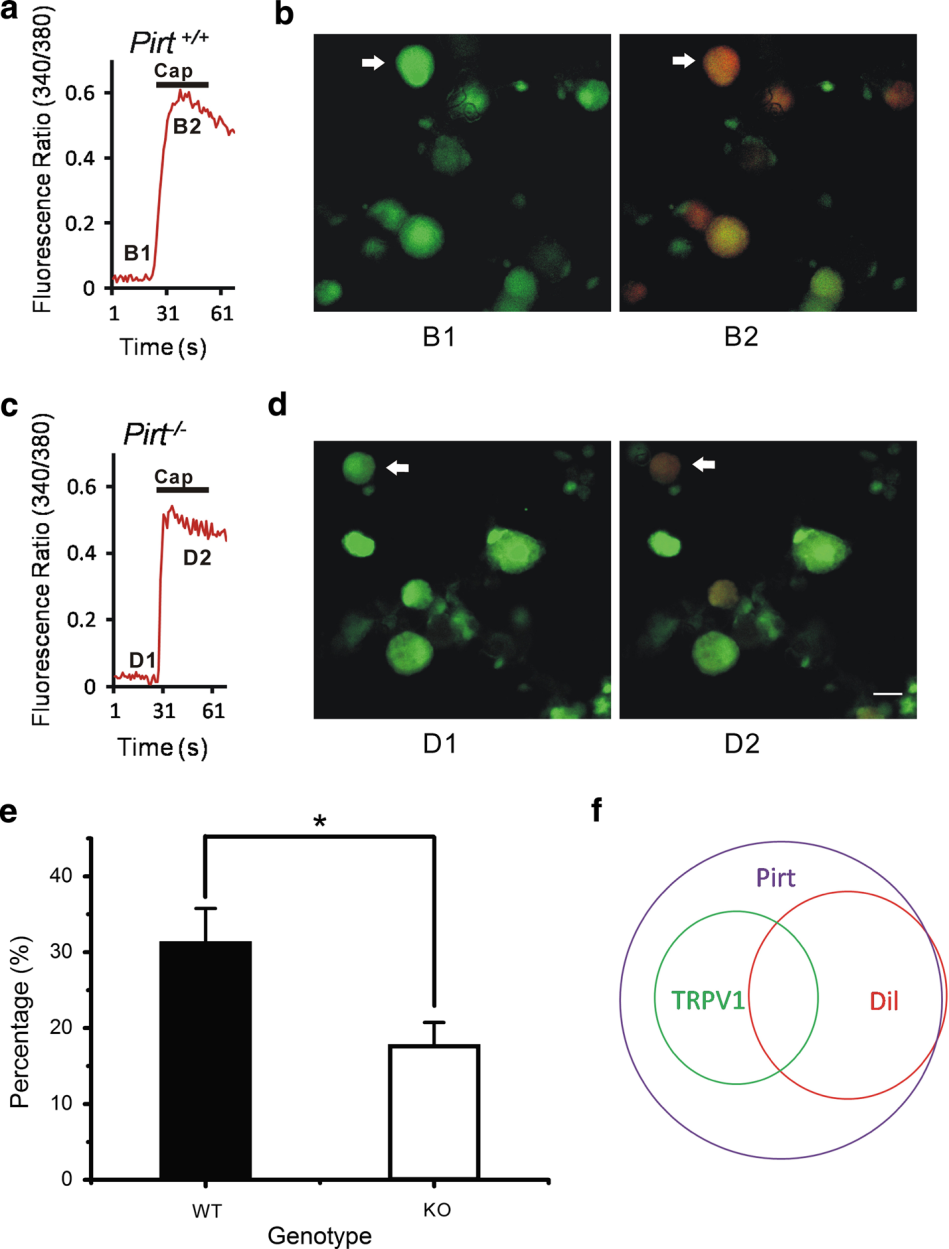
Dil-labeled DRG neurons respond to capsaicin. **a** Representative capsaicin-induced Ca^2+^ trace in DRG neuron from *Pirt* ^+*/*+^ mouse. **b** Images of DRG neurons from *Pirt* ^+*/*+^ mice before (*B1*) and after (*B2*) capsaicin treatment (5 μM). **c** Representative capsaicin-induced Ca^2+^ trace in DRG neuron from Pirt^+/+^ mouse. **d** Images of DRG neurons from Pirt^−/−^ mice before (*D1*) and after (*D2*) capsaicin. *Scale bar* 20 µm. **e** The proportion of DRG neurons from *Pirt* ^+*/*+^ and *Pirt* ^−*/*−^ mice that responded to capsaicin (% of total cells, *P < 0.05, n = 3/group). **f** 382 of 816 accounted *Pirt* ^+*/*+^ positive cells were Dil-labeled cells (46 %). 120 of 382 Dil-labeled cells (31 %) responded to capsaicin. 204 of 816 *Pirt* ^+*/*+^ neurons (25 %) responded to capsaicin

To further investigate the involvement of Pirt in UCP, we conducted a Ca^2+^ imaging study to compare the capsaicin-evoked response of Dil-labeled DRG neurons from *Pirt*^+*/*+^ and *Pirt*^−*/*−^ mice (Fig. 5a–d). The proportion of Dil-labeled DRG neurons responding to capsaicin in *Pirt*^+*/*+^ mice was significantly higher (31.4 ± 4.4 %) than the proportion in *Pirt*^−*/*−^ mice (17.8 ± 3.0 %, *P* < 0.05, n = 3 animals/group, Fig. 5e). In *Pirt*^+*/*+^ mice, 46 % (382 of 816) of Pirt-positive cells were Dil-labeled cells, in which 31 % (120 of 382) responded to capsaicin (Fig. 5f). By contrast, in *Pirt*^−*/*−^ mice, only 25 % (204 of 816) of Dil-labeled neurons responded to capsaicin.

## Discussion

Pirt is a phosphoinositide-binding protein that functions as a key regulatory subunit of the TRPV1 channel, which may be involved in visceral pain [14]. In the present study, we investigated the role of Pirt in the perception of UCP in female mice. We found that—after a 6-day period of estradiol priming and an oxytocin challenge—*Pirt*^**−/−**^ mice exhibited markedly reduced writhing behavior than their *Pirt*^+*/*+^ counterparts. In uterus, Pirt-expressing nerve terminals were limited to the myometrium. Importantly, Pirt-expressing DRG neurons innervating the uterus could respond to capsaicin. Furthermore, the proportion of DRG neurons responding to capsaicin was reduced in *Pirt*^−*/*−^ mice compared to that in *Pirt*^+*/*+^ mice. Altogether, our findings suggest that Pirt contributes to UCP.

Sex hormones play an important role in visceral pain hypersensitivity [21, 22]. In animal models of uterine contraction, estrogen-induced hyperalgesia was associated with increased TRPV1 expression in primary sensory neurons innervating the uterus [10], which showed that afferent responses to cervical distension were reduced by the antagonist of capsaicine (capsaizepine) in estrogen-treated animals [11]. However, estrogen did not affect the expression of Pirt. Based on these findings, we administered the estrogen to female mice for 6 days to prime the induction of pain hypersensitivity to uterine contractions; then, we administered the oxytocin on day 7 to evoke an UCP, as shown previously [18, 19]. Writhing behavior—which may indicate the presence of visceral pain—was less evident in *Pirt*^−/−^ mice than in *Pirt*^+*/*+^ mice. These observations suggest an involvement of Pirt in UCP. Interestingly, the writhing response was manifested in two phases after oxytocin injection, similar to that of formaldehyde-induced inflammatory pain [23, 24]. Although the mechanisms remain unclear, we postulate that the first phase may result from a direct action of oxytocin on the uterus, while the second phase may involve a sensitization of dorsal horn neurons and the release of algogenic factors after uterine contraction.

Pirt is expressed in most nociceptive DRG neurons, including TRPV1-positive cells. Previously, it was reported that *Pirt*^−*/*−^ mice show an impaired pain response to noxious heat stimuli and capsaicin [14]. Interestingly, *Pirt*^−*/*−^ mice also exhibited decreased responses to cold stimuli, suggesting that Pirt may also regulate cold-receptor TRPM8 function [15]. The pain originating from labor and gynecological disorders results primarily from distension of the uterine cervix and lower uterine segment [2, 10, 11]. We show here for the first time that Pirt-expressing nerve terminals innervate the myometrium of the uterus, providing a physiological basis for its involvement in UCP. There have been indications in the literature that female reproductive tract tissues (cervix and caudal uterus), as well as other abdominal organs such as the bladder and distal colon, are innervated by DRG neurons in T10–L2 segments [10, 25]. Visceral pain from pancreas, bladder, and colon has been proposed to be mediated by non-peptidergic, small-diameter DRG neurons [25, 26]. Our current findings support this point of view. Additionally, we show for the first time that DRG neurons that innervate the uterus are entirely Pirt-positive. Therefore, our studies support the concept that Pirt-expressing DRG neurons constitute a critical component of the pain transduction pathway involved in UCP.

TRPV1 can be activated by noxious heat stimuli and capsaicin [27]. Investigators have also recently analyzed the general architecture and physiological function of TRP-type channels [28, 29]. TRPV1-positive nerve fibers were found in the uterus, and TRPV1 was shown to be involved in UCP. For example, TRPV1 was suggested to play an important role in estrogen-induced sensitization of cervical afferents [10, 11]. Activation of TRPV1 receptors in nerve fibers that innervate the uterus also induced sensitization of the pelvic-urethra reflex [12]. Mechanical stimulation of somatosensory neurons induced physical interactions between the C-terminus of TRPV1 and microtubules of the cell [30], suggesting that TRPV1 has a role in transduction of mechanical stimuli. Pirt can bind to TRPV1 and positively regulate its function [14]. In our study, uterine DRG neurons from *Pirt*^+*/*+^ mice showed a higher response rate to capsaicin challenge than those from *Pirt*^−*/*−^ mice. As the binding of Pirt increased the excitability of TRPV1-expressing neurons [14], we reason that Pirt may play an important role in UCP through its interaction with TRPV1.

## Conclusion

Our findings indicate that Pirt plays a significant role in uterine contraction-induced pain (UCP) by comparison of behavior tests in *Pirt*^−*/*−^ mice and *Pirt*^+*/*+^ mice, the analysis of immunohistochemistry and calcium imaging on DRG neurons.

## Abbreviations

Pirt: phosphoinositide interacting regulator of transient receptor potential
UCP: uterine contraction-induced pain
TRPV1: transient receptor potential vanilloid channel 1
DRG: dorsal root ganglia
CNS: central nervous system
NF-200: neurofilament protein-200
IB_4_: plant lectin B_4_
Dil: 1,1′-Dioctadecyl-3,3,3′,3′-tetramethylindocarbocyanine perchlorate
OCT: optimum cutting temperature compound
